# Quality improvement in the determination of death by neurologic criteria around the world

**DOI:** 10.1186/s13054-023-04373-1

**Published:** 2023-03-21

**Authors:** Ariane Lewis, Matthew P. Kirschen, Rafael Badenes

**Affiliations:** 1 Department of Neurology and Neurosurgery, NYU Langone Medical Center, New York, NY USA; 2 Department of Anesthesiology and Critical Care Medicine, The Children’s Hospital of Philadelphia, Perelman School of Medicine at the University of Pennsylvania, Philadelphia, PA USA; 3 Department of Anesthesiology and Surgical-Trauma Intensive Care, Hospital Clinic Universitari, Valencia, Spain; 4 Department of Surgery, Anesthesiology and Critical Care, School of Medicine, University of Valencia, Valencia, Spain

## Abstract

This article is one of ten reviews selected from the Annual Update in Intensive Care and Emergency Medicine 2023. Other selected articles can be found online at https://www.biomedcentral.com/collections/annualupdate2023. Further information about the Annual Update in Intensive Care and Emergency Medicine is available from https://link.springer.com/bookseries/8901.

## Introduction

Public trust in the determination of brain death/death by neurologic criteria is contingent upon prevention of false positive determinations [1]. Unfortunately, although brain death/death by neurologic criteria is accepted throughout much of the world as equivalent to death by circulatory–respiratory criteria, numerous studies have reported variability across national and hospital policies, practice, and documentation for brain death/death by neurologic criteria determination [2–9]. Most recently, a 2020 study by Lewis et al. demonstrated inconsistency across 78 national policies [2]. This variability has the potential to lead to inaccurate brain death/death by neurologic criteria determinations.

The World Brain Death Project (WBDP), an international consensus statement, establishes minimum requirements for brain death/death by neurologic criteria determination in an effort to facilitate consistency, both within and between countries [10]. The WBDP was endorsed by five world federations—(1) the World Federation of Critical Care Nurses, (2) the World Federation of Intensive and Critical Care, (3) the World Federation of Neurology, (4) the World Federation of Neurosurgical Societies and (5) the World Federation of Pediatric Intensive and Critical Care Societies. The intent of the WBDP is for its minimum requirements for brain death/death by neurologic criteria determination to guide revision or creation of national policies for brain death/death by neurologic criteria determination around the world, after which hospital policies can be written or revised to reflect national policies. However, the WBDP questions what quality improvement measures can be put into place to ensure consistent and thorough brain death/death by neurologic criteria determination and eliminate the risk of false positive determinations [11].

Quality improvement efforts to promote accuracy and consistency in brain death/death by neurologic criteria determination must focus on updating national and hospital policies (which describe what clinicians should do) and addressing the discrepancy between policies and clinical practice (what clinicians actually do). This requires (1) medical society or governmental healthcare organizations to (a) align national brain death/death by neurologic criteria policies with the minimum requirements outlined by the WBDP and (b) facilitate regulation of hospital policies on brain death/death by neurologic criteria determination and credentialing of clinicians involved in brain death/death by neurologic criteria determination; (2) hospitals to ensure their policies are consistent with national policies; and (3) clinicians to be educated about the nuances of brain death/death by neurologic criteria determination [1].

The WBDP recommends that clinicians involved in brain death/death by neurologic criteria determination should (1) be licensed, (2) have completed training, and (3) be trained in brain death/death by neurologic criteria determination and in counseling families at the end-of-life [10]. The best way to evaluate and validate training programs and clinician competence in brain death/death by neurologic criteria determination is unknown. However, it is imperative that clinicians involved in brain death/death by neurologic criteria determination are familiar with conditions that could confound the evaluation (i.e., evaluate prerequisites for brain death/death by neurologic criteria determination), and with performance of the clinical examination and apnea test and the appropriate use of ancillary testing to prevent false positive brain death/death by neurologic criteria determinations. The WBDP statement includes a thorough review of this information including a checklist and flow diagram [10].

The goal of this chapter is to provide quality improvement guidance to prevent false positive brain death/death by neurologic criteria determinations and ensure patient safety during brain death/death by neurologic criteria determination. The chapter addresses: (1) brain death/death by neurologic criteria mimics, (2) confounders that interfere with the clinical brain death/death by neurologic criteria evaluation, (3) prevention of complications during the apnea test, (4) ancillary testing confounders and limitations, and (5) special considerations for brain death/death by neurologic criteria determination in pediatric patients.

## Brain Death/Death by Neurologic Criteria Mimics

The WBDP specifies that brain death/death by neurologic criteria determination requires identification of “an established neurological diagnosis, the nature and severity of which is capable of resulting in the irreversible loss of the capacity for consciousness, the irreversible loss of all brainstem reflexes and the irreversible loss of the ability to spontaneously breathe in the face of a carbon dioxide and acidosis challenge” [10]. Diagnoses that most often lead to brain death/death by neurologic criteria include hypoxic ischemic brain injury, stroke (ischemic stroke, subarachnoid hemorrhage, or intracerebral hemorrhage) and traumatic brain injury [12]. Other diagnoses that can lead to brain death/death by neurologic criteria include infections of the central nervous system (meningitis, encephalitis, abscesses), neoplasms, obstructive hydrocephalus, and edema secondary to severe metabolic derangements, such as acute hyponatremia, hyperammonemia or liver failure.

It is important to recognize there are a number of potentially reversible conditions that mimic brain death/death by neurologic criteria. Because of this, if the etiology of a patient’s neurologic state is unknown, or any of the conditions that can mimic brain death/death by neurologic criteria may be present, clinicians should not perform an evaluation for brain death/death by neurologic criteria (Table 1).

**Table 1:** Mimics of brain death/death by neurologic criteria [10]

Condition
Botulism
Cervical cord injury
Fulminant Guillain–Barré syndrome
Hypokalemia
Leptomeningeal carcinomatosis
Organophosphate poisoning
Rabies encephalitis
Snake bite

### Confounders That Interfere with the Clinical Brain Death/Death by Neurologic Criteria Evaluation

Even if a patient has a catastrophic brain injury from an etiology that can lead to brain death/death by neurologic criteria, there are a number of confounding conditions that have the potential to interfere with the brain death/death by neurologic criteria evaluation and could make a person appear as though they fulfil criteria, when they in fact do not (Table 2) [10, 13]. The WBDP defines “confounders” as “circumstances during which a diagnostic test or clinical evaluation may become unreliable and require repetition over time or application of an alternative test” [10]. The WBDP provides guidance about how to minimize the likelihood that confounders will lead to a false positive brain death/death by neurologic criteria determination.

**Table 2:** Confounding factors for the clinical brain death/death by neurologic criteria evaluation (from [13] with permission)

Disease process	Possible exam components confounded
Hypothermia	Complete exam
Muscular paralysis	Complete exam
Sedation/analgesia	Complete exam
Hypoxia	Complete exam
Hypotension	Complete exam
Hypoglycemia	Complete exam
Endocrine or metabolic abnormality	Complete exam
Basal skull fracture with hemotympanum	Oculovestibular reflex
Facial trauma	Pupillary response, oculovestibular, and oculocephalic reflexes, cranial pain response
Pulmonary injury/disease	Apnea test
Cervical spine injury	Corporal pain response, apnea test
Anophthalmia	Pupillary, corneal, oculovestibular, and oculocephalic reflexes

#### Hypothermia

Hypothermia can temporarily blunt brain activity leading to reversible coma, brainstem areflexia, and inability to breathe spontaneously [14]. Hypothermia may be induced environmentally or intentionally for therapeutic benefit through use of targeted temperature management (TTM) or may result from severe brain injury due to loss of sympathetic regulation and catecholamine release (41–100% of patients develop hypothermia in the setting of brain death/death by neurologic criteria) [15]. Despite this, Lewis et al. found that only 78% of national brain death/death by neurologic criteria policies addressed the minimum temperature for brain death/death by neurologic criteria determination, and it ranged from 32 to 36 °C [2]. Additionally, only 10% of national policies included information about brain death/death by neurologic criteria determination after hypothermia and only two national policies clearly specified the minimum duration to observe patients for neurologic recovery after rewarming before conducting the evaluation for brain death/death by neurologic criteria (both advised waiting at least 24 h after rewarming).

To ensure temperature does not confound brain death/death by neurologic criteria determination, the WBDP suggests a minimum core temperature of 36 °C (defined by esophageal, bladder, rectal, or central venous or arterial catheter temperature measurement) [10]. The WBDP advises use of a warming blanket, an automated temperature regulation device, a thermal mattress, warmed fluids, and/or warmed oxygen to achieve this temperature, if needed. The WBDP also provides detailed guidance about timing of the brain death/death by neurologic criteria evaluation after rewarming. This includes waiting a period of at least 24 h after rewarming to observe for neurologic recovery and a low threshold to perform ancillary testing to evaluate brain blood flow in addition to completion of the clinical brain death/death by neurologic criteria examination and apnea test.

#### Muscle Paralysis

Muscle paralysis due to pharmacological paralytics or a neuromuscular disorder can interfere with the assessment for irreversible loss of brain-mediated motor responses, brainstem reflexes and the ability to breathe spontaneously. However, Lewis et al. found that only 63% of national brain death/death by neurologic criteria policies noted that the effects of pharmacological paralytics must be excluded prior to brain death/death by neurologic criteria determination [2]. The WBDP recommends use of a train-of-four stimulator or demonstration of the presence of deep tendon reflexes prior to performance of an evaluation for brain death/death by neurologic criteria [10]. The WBDP also notes that ancillary testing should be performed in addition to completion of the clinical examination and apnea test for patients with a severe neuromuscular disorder, like amyotrophic lateral sclerosis, given that lack of motor responses in these patients cannot definitively be attributed solely to their catastrophic brain injury.

#### Sedation/Analgesia

Sedation and analgesia can depress the central nervous system (CNS), as can a number of other medications, including antibiotics, baclofen, bupropion, tricyclic antidepressants, and valproic acid [13]. Clearance of these medications can be delayed in the setting of renal or hepatic dysfunction, obesity or hypothermia. Although Lewis et al. found that the majority of national brain death/death by neurologic criteria policies (82%) noted the need to exclude the effects of drugs or medications that depress the CNS before conducting the evaluation for brain death/death by neurologic criteria, this was not uniformly specified (51% mentioned sedation and 31% mentioned analgesia) [2]. Pharmacological derangements were included as an indication for ancillary testing in 36% of national brain death/death by neurologic criteria policies.

The WBDP recommends excluding the effects of drugs or medications prior to evaluation for brain death/death by neurologic criteria (1) using a toxicology screen if there is concern for toxic exposure; (2) via measurement of drug levels, if able, to ensure they are within or below the therapeutic range and are not felt to have the 146 potential to confound the evaluation; or (3) by ensuring five elimination half-lives have passed, assuming normal hepatic and renal function [10]. If there is concern that drug elimination cannot be confirmed prior to evaluation for brain death/death by neurologic criteria, ancillary testing should be performed in addition to the clinical examination and apnea test.

#### Hypoxia

Hypoxic-ischemic brain injury can temporarily blunt brain activity, but Lewis et al. found that very few national brain death/death by neurologic criteria policies (17%) specified the need for an observation period after hypoxic-ischemic brain injury before evaluation for brain death/death by neurologic criteria [2, 16]. These policies all noted it was necessary to observe for at least 24 h after resuscitation to confirm there had been no neurologic recovery before initiating the evaluation for brain death/death by neurologic criteria [2]. Additionally, 7% of national brain death/death by neurologic criteria policies recommended performance of ancillary testing for patients with hypoxic-ischemic brain injury.

The WBDP recommends that patients should be observed for at least 24 h after hypoxic-ischemic brain injury, with consideration for ancillary testing in the setting of hypothermia after resuscitation following hypoxic-ischemic brain injury (as discussed above) [10].

#### Hypotension

Hypotension occurs in 65–97% of patients with catastrophic brain injury due to loss of hypothalamic function, loss of central vasomotor regulation of sympathetic tone, decreased cardiac contractility, hypovolemia, or other causes of shock [15]. Although systemic hypotension results in brain hypoperfusion, which can cause transient reversible loss of brain function, Lewis et al. found that only 44% of national brain death/death by neurologic criteria policies addressed the minimum blood pressure for brain death/death by neurologic criteria determination [2]. Some policies required normotension or absence of shock or hemodynamic instability, whereas other policies specified the minimum acceptable blood pressure (mean arterial pressure [MAP] >60 mmHg, MAP >70 mmHg, systolic blood pressure [SBP] > 90 mmHg, SBP > 100 mmHg, or other threshold). Cardiovascular instability was noted to be an indication for ancillary testing in 7% of national brain death/death by neurologic criteria policies.

The WBDP suggests a minimum MAP of 60 mmHg or SBP of 100 mmHg (and age appropriate targets in pediatric patients) prior to evaluation for brain death/death by neurologic criteria [10].

#### Hypoglycemia or Other Endocrine or Metabolic Abnormality

Hypoglycemia can lead to reversible loss of brain function, but there is no established lower limit of glucose for brain death/death by neurologic criteria determination [10, 17]. However, in a survey of 84 physicians in the Neurocritical Care Society, Lerner et al. found that 33% of respondents reported that their institution stipulated the minimum glucose for evaluation for brain death/death by neurologic criteria [17]. Respondents also commented on the laboratory thresholds they personally found acceptable for brain death/death by neurologic criteria determination. The median lower glucose threshold accepted among respondents was 60 mg/dl (interquartile range (IQR) 50–69 mg/dl).

Other endocrine or metabolic abnormalities could also impact the evaluation for brain death/death by neurologic criteria. Lewis et al. found that the need to rule out electrolyte, acid/base or endocrine derangements before brain death/death by neurologic criteria determination was addressed in 72% of national policies, though very few provided specific values (8% indicated specific electrolyte values, 5% included a specific acid/base threshold, and 1% noted specific endocrine values) [2]. Metabolic derangements were noted to be an indication for ancillary testing in 19% of national brain death/death by neurologic criteria policies.

The aforementioned survey by Lerner et al. demonstrated that there were varying perspectives on the specific metabolic values that preclude brain death/death by neurologic criteria determination. In addition to glucose, arterial pH and sodium were considered the most important variables to check [17]. The median personal lower and upper pH thresholds were 7.25 (IQR 7.1–7.3) and 7.55 (IQR 7.5–7.6), respectively, while the median personal lower and upper sodium thresholds were 125 mEq/l (IQR 120–130 mEq/l) and 160 mEq/l (IQR 155–170 mEq/l), respectively.

The WBDP recommends correction of severe metabolic, acid–base, and endocrine derangements, when feasible, and performance of ancillary testing to augment the clinical evaluation if these derangements cannot be corrected, but does not provide specific lower or upper limits for laboratory values [10].

#### Basal Skull Fracture with Hemotympanum

Lewis et al. noted variable guidance across national brain death/death by neurologic criteria policies about whether the oculovestibular reflex can be tested in the setting of basal skull fracture; 19% indicated a ruptured eardrum precluded the assessment, 9% indicated a ruptured eardrum does not preclude the assessment, and 5% specified that a skull fracture could blunt the response [2]. The need for ancillary testing in patients with a ruptured eardrum was required in 7% of national brain death/death by neurologic criteria policies.

The WBDP recommends ancillary testing in addition to completion of the clinical examination and apnea test for patients with a fracture of the base of the skull or petrous temporal bone given the potential for this type of injury to make the oculovestibular reflex unreliable on the side of the fracture [10]. It also notes that presence of a ruptured tympanic membrane does not negate evaluation of the reflex, but that this could lead to an increased risk of meningitis.

#### Facial Trauma

Lewis et al. found that national brain death/death by neurologic criteria policies provided inconsistent guidance about the ability to perform a complete brain death/death by neurologic criteria evaluation in patients with facial trauma; 25% noted this was an indication for ancillary testing [2].

The WBDP advises that facial trauma could interfere with assessment of the pupillary, oculovestibular, and oculocephalic reflexes or the motor response of the face, necessitating ancillary testing in addition to completion of the clinical examination and apnea test [10].

#### Pulmonary Injury/Disease

Acute lung injury or chronic pulmonary disease has the potential to interfere with the apnea test [10]. Lewis et al. found that 50% of national brain death/death by neurologic criteria policies noted that inability to complete the apnea test warranted ancillary testing [2]. Safety considerations for performance of the apnea test are discussed in detail below.

#### Cervical Spine Injury

Cervical spine injury has the potential to interfere with evaluation of motor responses, the oculocephalic reflex, the cough reflex, and the apnea test [10]. Lewis et al. found that 32% of national brain death/death by neurologic criteria policies indicated that cervical spine injury should be ruled out before assessment of the oculocephalic reflex, 3% indicated this should be ruled out before assessment of the cough reflex, and 5% indicated this should be ruled out before the apnea test; 9% specified that ancillary testing should be performed in patients with cervical spine injury [2].

The WBDP advises that the oculocephalic reflex should not be tested in patients with a cervical spine injury, but that if the oculovestibular reflex is performed and there are no extraocular movements, ancillary testing is not required as long as the rest of the clinical examination and the apnea test are completed and consistent with brain death/death by neurologic criteria [10]. However, it also notes that high spinal cord injuries can impact the efferent limb of the cough reflex and the apnea test and therefore recommends performance of ancillary testing.

#### Anophthalmia

In the setting of anophthalmia, the pupillary, corneal, oculovestibular, and oculocephalic reflexes on the side without an eye cannot be evaluated. Lewis et al. found that 14% of national brain death/death by neurologic criteria policies specified that ancillary testing should be performed in patients with only one eye [2].

The WBDP recommends ancillary testing in addition to completion of the clinical examination and the apnea test for patients with anophthalmia [10].

### Considerations for Performance of the Clinical Examination

After ruling out the aforementioned mimics and confounders for the evaluation of brain death/death by neurologic criteria, the clinical examination can be performed. The steps for performance of the clinical examination for brain death/death by neurologic criteria determination, responses consistent with brain death/death by neurologic criteria, and special considerations enumerated by the WBDP are summarized in Table 3 [10].

**Table 3:** Technique and considerations for the performance of the clinical brain death/death by neurologic criteria (BD/DNC) examination (from [10] with permission)

Component	Test	Response consistent with BD/DNC	Considerations
Coma	Apply maximal external stimulation (including noxious visual, auditory, and tactile stimulation) to assess for arousal or awareness	There is no evidence of arousal or awareness to maximal external stimulation (including noxious visual, auditory, and tactile stimulation)	
Motor responses of the face and limbs	Apply deep pressure to all of the following: 1. The condyles at the level of the temporomandibular joints 2. The supraorbital notch bilaterally the sternal notch 3. All four extremities, both proximally and distally Insert a cotton swab on a stick in each nostril to perform “nasal tickle” testing	Noxious stimuli should not produce grimacing, facial muscle movement, or a motor response of the limbs other than spinally mediated reflexes Noxious stimuli above the foramen magnum should not produce any movement in the face or body. Noxious stimuli below the foramen magnum should not produce any movement in the face but may elicit spinally mediated peripheral motor reflexes	The clinical differentiation of spinal from brain-mediated motor responses requires expertise. Consultation with an experienced practitioner is recommended if the origin of a response is unclear. Alternatively, if interpretation is unclear, ancillary testing is recommended Ancillary testing is recommended if a person has a preexisting severe neuromuscular disorder, such as amyotrophic lateral sclerosis or a preexisting severe sensory neuropathy Ancillary testing is not required if a person does not have all four limbs; absence of a limb does not preclude motor testing to pain on that side of the body Severe facial trauma or swelling may preclude evaluation of facial motor response, so ancillary testing is recommended in this setting
Pupillary reflex	Shine a bright light into each of the person’s eyes, looking for pupillary constriction and measuring the diameter of the pupils (use of a magnifying glass and/or pupillometer is suggested)	There should be absence of ipsilateral and contralateral pupillary response, with pupils fixed in a midsize or dilated position (≈4–6 mm), in both eyes	Constricted pupils are not consistent with BD/DNC and suggest the possibility of drug intoxication or locked-in syndrome Pupils can be any shape (round/oval/irregular) Corneal trauma or prior ophthalmic surgery may influence pupillary reactivity and preclude adequate evaluation, necessitating ancillary testing Ocular instillation of drugs may artificially produce transiently nonreactive pupils In the setting of anophthalmia or inability to see the pupils, ancillary testing is recommended
Oculocephalic (OCR) and oculovestibular (OVR) reflexes	OCR: Rotate the head briskly horizontally to both sides. There should be no movement of the eyes relative to head movement. Testing vertically is optional OVR: Examine the auditory canal for patency and an intact tympanic membrane. Elevate the head to 30° to place the horizontal semicircular canals in the correct vertical position. Irrigate with at least 30 ml of ice water for at least 60 s using a syringe or a syringe attached to a catheter placed inside the canal. Test both sides separately, with a 5-min interval between to allow the endolymph temperature to equilibrate	There should be absence of extraocular movements. Detection of any extraocular movements is not compatible with BD/ DNC	Confirm the integrity of the cervical spine before proceeding with the OCR test. If the OCR cannot be performed, but the OVR is performed and there are no extraocular movements, ancillary testing is not required Ensure the integrity of the tympanic membrane. Presence of a ruptured tympanic membrane does not negate the clinical testing but may risk introducing infections in the ear A fracture of the base of the skull or petrous temporal bone may obliterate the response on the side of the fracture, and ancillary testing is recommended in this instance Severe orbital or scleral edema or chemosis may affect the free motion of the globes, and ancillary testing is recommended in this instance In the setting of anophthalmia, ancillary testing is recommended
Corneal reflex	Touch the cornea of both eyes with a cotton swab on a stick at the external border of the iris, applying light pressure and observing for any eyelid movement	No eyelid movement should be seen	Care should be taken to avoid damaging the cornea In the setting of anophthalmia, severe orbital edema, prior corneal transplantation, or scleral edema or chemosis, ancillary testing is recommended
Gag and cough reflexes	Gag reflex: stimulate the posterior pharyngeal wall bilaterally with a tongue depressor or suction catheter Cough reflex: stimulate the tracheobronchial wall to the level of the carina with deep endotracheal placement of a suction catheter	Absence of gag and cough	The efferent limb for the cough reflex includes the phrenic nerve, which may be injured in persons with high cervical cord injuries, so ancillary testing is recommended in this setting

#### Apnea Test Safety Considerations

Safety considerations for performance of the apnea test are discussed here, but a detailed discussion of the procedure for performance of the apnea test can be found in the WBDP summary and supplemental documents [10]. The WBDP advises that the brain death/death by neurologic criteria evaluation should not begin until it has been determined that the patient is not taking any spontaneous breaths when the ventilator is in spontaneous breathing mode and that the apnea test should only be conducted after the clinical examination has been completed and found to be consistent with brain death/death by neurologic criteria. Further, the WBDP recommends that ventilator requirements and pulmonary status be assessed prior to the apnea test to determine whether the patient is likely to tolerate the test.

Although the apnea test is generally considered safe, induction of a hypercarbic acidemic state can lead to complications (Table 4). Reviews of the literature have demonstrated that the apnea test needs to be aborted due to hemodynamic instability in 1–20% of patients, particularly those of younger age, with lower pre-test pH, hypotension, a high arterial-alveolar gradient, or polytrauma [10, 18, 19]. However, the risks of the apnea test can be minimized through strict adherence to guidance on ensuring prerequisites are met prior to the apnea test.

**Table 4:** Apnea test safety considerations [10, 15, 18]

Complication	Prevention techniques
Hypotension	Ensure MAP > 60 mmHg or SBP > 100 mmHg before testing is initiated Use fluids/vasopressors/inotropes as needed Ensure euvolemia/hypervolemia prior to testing Ensure temperature is > 36 °C Abort testing for MAP < 60 mmHg or SBP < 100 mmHg
Hypoxemia	Preoxygenate with 100% oxygen for 10-min prior to testing Consider checking arterial blood gas to ensure PaO_2_ > 200 mmHg Consider use of CPAP or a PEEP valve Abort testing for sustained desaturation < 85%
Pneumothorax, pneumomediastinum, and pneumoperitoneum	Ensure insufflation catheter tip is in the distal third of the endotracheal tube Ensure insufflation catheter is not too wide compared to the diameter of the endotracheal tube Minimize oxygen flow rate through insufflation catheter
Arrhythmia/cardiac arrest	Prevent hypotension and hypoxemia Correct electrolyte derangements Ensure temperature is > 36 °C

*MAP* Mean arterial pressure, *SBP* Systolic blood pressure, *CPAP* continuous positive airway pressure, *PEEP* Positive end-expiratory pressure

Nonetheless, because of the potential for complications during the apnea test, the WBDP notes that it should be performed by clinicians with experience in cardiopulmonary resuscitation (CPR) [10]. The WBDP advises that if the apnea test is aborted, ancillary testing should be performed or the apnea test should be repeated after waiting additional time for resolution of hemodynamic instability. If the apnea test is aborted due to hypoxemia, consideration can be given to repeating it after improvement in pulmonary dysfunction (perhaps via performance of recruitment maneuvers) or using continuous positive airway pressure (CPAP) or a positive end-expiratory pressure (PEEP) valve to minimize the risk of hypoxemia or induction of hypercarbia with carbogen to reach the target PaCO_2_ and pH faster.

#### Hypotension

Patients may develop hypotension during the apnea test as a result of either the acidosis itself or preexisting conditions; these include arteriolar vasodilation, decreased preload, or depressed myocardial contractility [10, 18]. A review of complications during the apnea test by Busl et al. found that the incidence of hypotension during the apnea test was 7–39% [18]. Despite this, only 14% of national brain death/death by neurologic criteria policies recommended aborting the apnea test if hypotension occurred [2].

To minimize the risk of hypotension during an apnea test and ensure the evaluation is not impacted by hypoperfusion of the medullary chemoreceptors, the WBDP suggests a minimum MAP of 60 mmHg or SBP of 100 mmHg (and age appropriate targets in pediatric patients) be maintained for the apnea test through use of fluids, vasopressors, or inotropes as needed [10]. Further, it recommends aborting the test for MAP < 60 mmHg or SBP < 100 mmHg.

#### Hypoxemia

The apnea test can lead to hypoxemia due to atelectasis [10, 18]. Busl et al. found that the incidence of hypoxemia during the apnea test was 4–6% [18]. Despite this, only 23% of national brain death/death by neurologic criteria policies recommended that the apnea test be aborted in case of hypoxemia [2].

To decrease the risk of hypoxemia during an apnea test, the WBDP suggests that patients be preoxygenated with 100% oxygen for at least 10-min prior to the apnea test and that use of CPAP or a PEEP valve be considered to prevent atelectasis [10]. Some national brain death/death by neurologic criteria policies specified that the apnea test should only be performed after an arterial blood gas demonstrates the PaO_2_ is ≥ 200 mmHg [2]. The WBDP also recommends aborting the apnea test for sustained desaturation < 85% [10].

#### Pneumothorax, Pneumomediastinum and Pneumoperitoneum

Barotrauma can lead to pneumothorax, pneumomediastinum or pneumoperitoneum during the apnea test [10, 18]. This can develop due to a number of mechanisms. If an insufflation catheter is used and the catheter is placed too deep, there can be tracheal perforation or if the catheter is too wide, gas flow can be obstructed. Secretions can also obstruct oxygenation or gas outflow. Finally, barotrauma can develop if oxygen flow is too high. Busl et al. reported that barotrauma during the apnea test was very rare [18]. To prevent these complications, it has been suggested that the oxygen insufflation catheter tip be in the distal third of the endotracheal tube, the catheter diameter be < 50–70% of the endotracheal tube diameter, and hyperoxygenation be avoided (< 6 l/min is generally advised) [2, 10].

#### Arrhythmia/Cardiac Arrest

An arrhythmia or cardiac arrest can occur during the apnea test as a consequence of hypotension, hypoxemia or acidosis. Busl et al. found that the incidence of arrhythmia during the apnea test was 1% and the incidence of cardiac arrest was < 1% [18]. The recommendation to abort the apnea test due to a dysrhythmia was only mentioned in 21% of national brain death/death by neurologic criteria policies [2]. The WBDP recommends aborting the apnea test if an unstable arrhythmia occurs [10].

## Ancillary Testing Confounders/Limitations

Lewis et al. found variability in ancillary testing across national brain death/death by neurologic criteria policies; this included variability in (1) the accepted ancillary tests, (2) guidance on performance and interpretation of the tests, and (3) an explanation of the confounders/limitations associated with ancillary testing [2, 20]. Of the national brain death/death by neurologic criteria policies that included an assessment of brain blood flow, < 10% described confounders/limitations that could impact interpretation of the results [20]. Of the national policies that included electroencephalography (EEG) as an acceptable ancillary test, 20% noted the potential for artifact, 18% noted it could be affected by sedatives, 16% noted it could be affected by hypothermia, 11% noted it could be affected by metabolic derangements, and 11% noted it predominantly assessed the cortex.

The WBDP recommends ancillary testing is required if the clinical examination and apnea test cannot be completed, confounding conditions cannot be resolved, or there is uncertainty about interpretation of spinal-mediated reflexes [10]. The WBDP includes three acceptable ways to evaluate for the absence of brain blood flow for the purposes of brain death/death by neurologic criteria determination: (1) digital subtraction angiography, (2) radionuclide angiography/scintigraphy, and (3) transcranial Doppler ultrasonography [10]. The WBDP suggests against routine use of EEG as an ancillary test since it does not provide information about brainstem 364 function, and advises that other tests to evaluate brain blood flow (e.g., computed 365 tomography angiography or magnetic resonance angiography) or electrical activity 366 (e.g., evoked potentials) should not be utilized. Confounders and limitations for each ancillary test are summarized in Table 5 [10, 23, 24].

**Table 5:** Ancillary testing confounders/limitations [10, 20–22]

Test	Confounder/limitation^a^	Sensitivity/specificity
*Brain blood flow*		
Computed tomography angiography	Requires transport to the scanner	52–97%/100%
	Image variability based on injection technique	
	Potential to miss a slow flow state	
	Risk of nephrotoxicity	
Digital subtraction angiography^b^	Requires transport to angiography suite	100%/100%
	Risk of nephrotoxicity	
	Image variability based on injection technique	
Magnetic resonance angiography	Requires transport to the scanner	93–100%/100%
	Image variability based on injection technique	
Radionuclide angiography^b^	Limited evaluation of brainstem	99%/56%
Radionuclide scintigraphy^b^	Requires transport to the scanner	Planar: 78%/100% SPECT: 88%/100%
	Planar imaging may have limited evaluation of brainstem	
Transcranial Doppler ultrasonography^b^	Potential for technical difficulties in performance	90%/98%
	Potential for lack of windows	
*Electrophysiological function*		
Electroencephalography	Risk of environmental artifact	53–80%/97%
	Confounded by sedation, hypothermia and toxic-metabolic derangements	
	Predominantly provides information about cortical function	
Evoked potentials-auditory	Can be absent in comatose patients with other intact brainstem reflexes	NA/NA
	Confounded by sedation, hypothermia, isolated eighth nerve and brainstem lesions	
	Only evaluates auditory pathways	
	Performance/interpretation may be limited by experience	
Evoked potentials-somatosensory	Can be absent in comatose patients with ongoing brain function	100%/78%
	Confounded by cervical spine injury, isolated brainstem lesions, sedation and hypothermia	
	Only evaluates somatosensory pathways	
	Performance/interpretation may be limited by experience	
Evoked potentials-visual	Can be absent in comatose patients with ongoing brain function	NA/NA
	Confounded by sedation, retinal or optic nerve lesions	
	Only evaluates visual pathways	
	Performance/interpretation may be limited by experience	

*NA* Not available

^a^Performance/interpretation of all ancillary tests may be limited by experience

^b^Accepted by the World Brain Death Project (WBDP)

### Special Considerations for Brain Death/Death by Neurologic Criteria Determination in Pediatric Patients

In some nations, brain death/death by neurologic criteria policies vary based on age to account for physiologic and anatomic differences in younger patients that necessitate a higher degree of conservatism [2, 20, 25]. For example, the USA has different brain death/death by neurologic criteria policies for adults and infants/children [26, 27]. Where pediatric specific guidance is provided, it addresses prerequisites for the brain death/death by neurologic criteria evaluation, the clinical examination, the apnea test, and ancillary testing [2].

First, drug metabolism can be different in pediatric patients compared to adults, so this should be accounted for when assessing fulfilment of prerequisites for brain death/death by neurologic criteria determination.

Second, serial examinations with a specified observation period between them to ensure there is no recovery of neurologic function, and/or a longer observation period prior to brain death/death by neurologic criteria determination, is sometimes required for pediatric patients [2]. This is particularly important for infants after cardiac arrest, since their brainstem may be more resistant to hypoxic-ischemic brain injury than older children and adults [25]. Infants with open fontanelles also may not have the same consequences of intracranial pressure and herniation as adults. While the WBDP suggests that a single evaluation is the minimum standard for brain death/death by neurologic criteria determination in adults, two clinical examinations and apnea tests are considered the minimum standard for brain death/death by neurologic criteria determination in pediatric patients [10]. Additionally, the WBDP recommends a “cautious approach with serial examinations and an adequate observation period” to minimize the risk of diagnostic error. However, the WBDP questions whether a single examination may be practical and safe for brain death/death by neurologic criteria determination in children [11].

Third, the WBDP recommends that tracheal insufflation should not be used for the apnea test in newborns, infants, and young children [10]. CPAP can be used in children to prevent atelectasis; this has a low adverse event rate [19]. The WBDP also suggests that ancillary testing be performed *in lieu* of the apnea test in patients with chronic hypoxemia due to cyanotic heart disease [10].

Finally, the WBDP recommends a radionuclide cerebral blood flow study as the preferred ancillary test and suggests that EEG is a valid ancillary study in infants and children and can be used in certain jurisdictions [10]. The WBDP also recommends that transcranial Doppler ultrasonography not be used in pediatric patients due to lack of data.

## Conclusion

Quality improvement in brain death/death by neurologic criteria determination is needed to prevent false positive determinations and subsequent loss of public trust in brain death/death by neurologic criteria. This improvement requires education of clinicians involved in brain death/death by neurologic criteria determination and revision of national and hospital brain death/death by neurologic criteria policies to meet the minimum requirements described by the WBDP [10]. Clinicians involved in brain death/death by neurologic criteria determination must be knowledgeable about (1) brain death/death by neurologic criteria mimics, (2) confounders that interfere with the clinical brain death/death by neurologic criteria evaluation, (3) prevention of complications during the apnea test, (4) ancillary testing confounders/limitations, and (5) special considerations for brain death/death by neurologic criteria determination in pediatric patients. Education about brain death/death by neurologic criteria determination can be found in the Neurocritical Care Society’s Brain Death Determination Course, an online continuing medical education module endorsed by the American Academy of Neurology, which reviews components of the evaluation, prerequisites, the clinical examination, the apnea test, ancillary testing, documentation, and communication [28, 29]. Finally, other institutional or national systems to prevent false positive brain death/death by neurologic criteria determinations include requiring credentialing of clinicians involved in brain death/death by neurologic criteria determination and oversight of hospital brain death/death by neurologic criteria policies by regulatory authorities [1, 30]. However, it is unknown what is the best (1) way to evaluate and validate the quality and efficacy of brain death/death by neurologic criteria training programs, (2) way to assess for clinician competence in brain death/death by neurologic criteria determination, and (3) means to certify and recertify clinicians for brain death/death by neurologic criteria determination [10]. Quality improvement research related to optimization of the accuracy and consistency of brain death/death by neurologic criteria determinations is needed.

## Data Availability

Not applicable.
